# Study on adsorption of hexavalent chromium by composite material prepared from iron-based solid wastes

**DOI:** 10.1038/s41598-023-27414-9

**Published:** 2023-01-04

**Authors:** Jiamin Qi, Bin Li, Pengxiang Zhou, Xintai Su, Di Yang, Jinxiong Wu, Zixuan Wang, Xiangjing Liang

**Affiliations:** 1 Faculty of Environmental Science and Engineering, Kunming University of Science and Technology, Kunming, 650500 China; 2 National-Regional Engineering Center for Recovery of Waste Gases from Metallurgical and Chemical Industries, Kunming, 650500 China; 3 School of Environment and Energy, Guangdong Provincial Key Laboratory of Solid Wastes Pollution Control and Recycling, South China University of Technology, Guangzhou, 510006 Guangdong China; 4 Yili Normal University, Yining, 835000 Xinjiang China; 5 Guangzhou Haitao Environmental Protection Technology Company Limited, Guangzhou, 510006 Guangdong China

**Keywords:** Ecology, Environmental sciences

## Abstract

A new adsorbent with chromium removal function was synthesized by carbon thermal method using iron-containing waste Fenton sludge and carbon-containing solid waste fly ash to treat high pH scoring wastewater generated from industrial processes. The results showed that the adsorbent used T = 273.15 K, pH = 10, t = 1200 min, C_0_ = 100 mg/L, had a removal rate of Cr(VI) of more than 80%, and the adsorption capacity could reach 393.79 mg/g. The characterization results show that the synthesized mesoporous nitrogen-doped composite material has a large specific surface area and mesoporous structure, and the surface of the material is rich in oxygen-containing functional groups and active sites. Compared with other studies, the adsorption capacity of the material is larger, which indicates that the removal effect of Cr(VI) in this study is better. The adsorption kinetic results show that the adsorption follows a pseudo second kinetic model, and the adsorption process is a chemisorption involving electron sharing or electron exchange. This experiment designed a simple method to synthesize mesoporous nitrogen-doped composites using industrial solid waste, with raw materials from cheap and easily available industrial solid waste, and solved the dual problems of heavy metals in wastewater and solid waste, providing a new idea for the resource utilization of Fenton sludge while not producing secondary pollution.

## Introduction

Chromium and its compounds are widely used in tanning, textiles, electroplating, dyes, fuels, and wood preservation^1^. The application of chromium and its compounds has inevitably caused a series of pollution while driving rapid economic growth^2^. Cr(VI) is mostly found in water, and it is a representative pollutant with high toxicity and mobility. Cr(VI) exists in different forms at different pH, with HCrO_4_^−^ dominating at low pH conditions and CrO_4_^2−^ at high pH conditions. Cr(VI) is 500 times more toxic than Cr(III), and it is one of the three internationally recognized carcinogenic metals. Chromium is a serious threat to aquatic organisms and human health, so chromium removal from wastewater is necessary and urgent^3^. Among the many chromium removal techniques, adsorption is now an efficient and economical solution due to its high removal rate, high regeneration potential, low initial cost, simple design, and ease of operation^4^. Various adsorbents such as metal oxides, activated carbon, and biomaterials are present in the market. Among these adsorbents, carbon materials are highly preferred due to the diversification of raw materials and low cost and are considered to be the most promising materials for the removal of heavy metals^5^. However, many adsorbents currently have certain drawbacks, such as poor adsorption capacity and slow adsorption rate, which limit the application and development of adsorbents and therefore correspond to the need to develop adsorbents with better adsorption capacity and lower production costs to treat chromium-containing wastewater^6^.

Wastes and by-products from industrial processes are considered as one the sources of low-cost adsorbents^7^. Fly ash can not only solve the waste pollution problem but also significantly reduce the cost of preparation of mesoporous nitrogen-doped composites. Fly ash is a type of industrial solid waste produced by the combustion of various organic and inorganic components in the coal-fired power generation process at temperatures ranging from 1200 to 1700 °C. The annual production of fly ash in the world is about 450 million tons, and the annual production in China is about 100 million tons. The current treatment method is mainly stockpiling, and improper treatment can cause air, water, and soil pollution, which is harmful to the environment and ecology^8^. Fenton sludge is a kind of hazardous waste produced by the Fenton process, which is mainly composed of iron ions and needs to be treated properly. It is mainly treated by incineration or landfill, but it will cause secondary pollution to the environment. It is considered to be a potential resource because of its high iron content, and many studies have been devoted to its conversion into resources for secondary utilization. Ye et al.^9^ used pyrolysis to convert Fenton sludge into magnetic sludge-based biochar, which was used as a catalyst to activate hydrogen peroxide for the removal of methylene blue from wastewater, and this study found that the catalytic capacity of the prepared catalyst could be maintained at 88.13% and was able to degrade 98.56% of 100 mg/L of methylene blue within 3 min, which was cost-effective, good and very friendly to the environment. Tong et al.^10^ used Fenton sludge to synthesize hydrogen amide carbon by a one-step hydrothermal method and used it to adsorb Pb^2+^ and found that its adsorption capacity could reach 359.83 mg/L with good results. This study provides a way for the resource utilization of fly ash and Fenton sludge. Conventional activated carbon synthesis uses carbon-rich feedstock for anaerobic carbonization, and solid waste-based mesoporous nitrogen-doped composites use carbon repeatedly heated to regenerate iron compounds and carbon-containing compounds^11^. The high cost and environmental risk issues limit the development of conventional methods. Mesoporous nitrogen-doped composites have become one of the research hotspots for the treatment of chromium-containing wastewater due to their low cost and environmental advantages. And Fenton sludge has a high iron content and can provide an iron source for the preparation of mesoporous nitrogen-doped composites^11–14^, while the fly ash used has a high carbon content and can provide a carbon source for the preparation of the material; therefore, it is theoretically possible to use Fenton sludge and fly ash to prepare mesoporous nitrogen-doped composites.

In this study, the mesoporous nitrogen-doped composite material was prepared by a simple carbothermal method using Fenton sludge solid waste from the Datansha sewage treatment plant in Guangzhou, China, and fly ash from the thermal power plant in Xinjiang, China, as the main raw materials. This study is aimed at industrial wastewater containing chromium with high pH. At present, most of the studies are focused on wastewater containing chromium with pH < 7. The research on high pH is less and the mechanism is not clear enough. In the process, the Fenton sludge is recycled to provide an iron element in the process of preparing the adsorbent, and the fly ash is a large industrial solid waste to provide carbon elements. Cr(VI) is a highly toxic heavy metal, which is widely used in the chemical industry. It is easy to cause cancer, teratogenesis, and mutagenesis and poses a serious threat to the animal, plant, and human health. Therefore, the adsorption capacity of the prepared adsorbent is measured as the target pollutant in the study. The effects of preparation conditions (such as the dosage of fly ash and Fenton sludge) on the adsorption performance of adsorbent and the adsorption performance of the adsorbent under different reaction conditions (such as pH, temperature, adsorption time, and initial concentration) were studied to determine the optimal conditions for Cr(VI) adsorption. And through a series of characterization means to explore the adsorption mechanism. This study not only can make the general industrial solid waste fly ash and hazardous solid waste Fenton sludge get resource utilization to reduce its pollution to the environment, but also can be used to prepare adsorption materials to remove Cr(VI) in chromium wastewater, solve the problem of heavy metals in solid waste and wastewater, thus will not produce secondary pollution, to achieve the purpose of waste treatment by waste.

## Results

### Material characterization results

**Figure 1:**
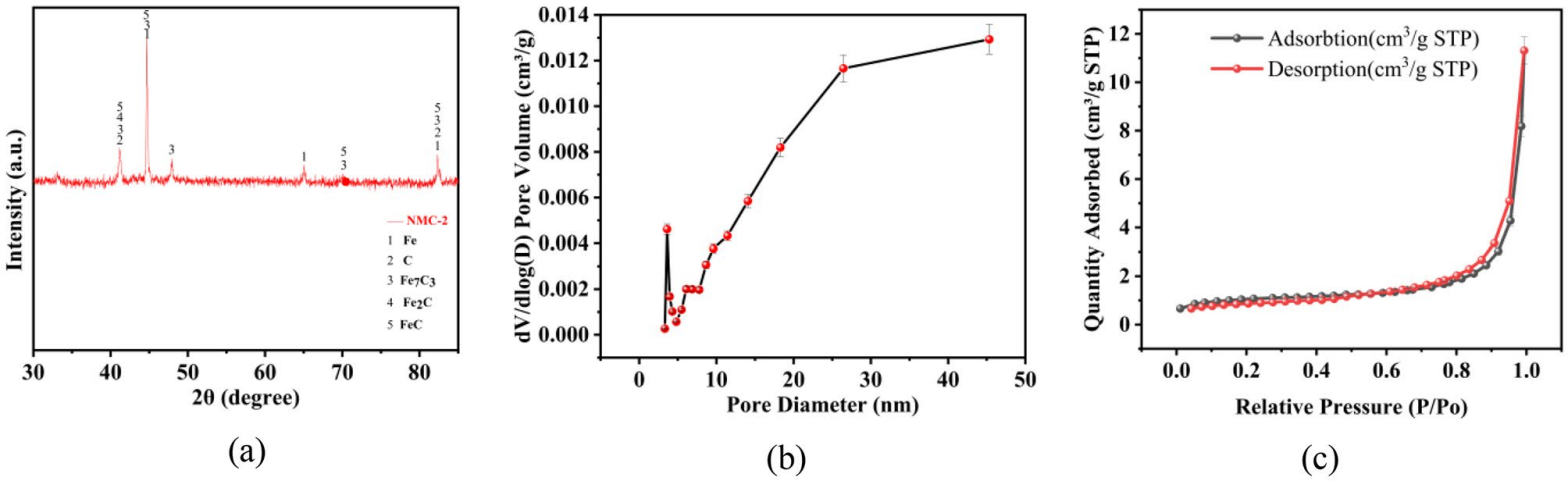
XRD and nitrogen adsorption and desorption tests on materials: (**a**) XRD pattern of NMC-2 adsorbent before adsorption, (**b**) pore size distribution of NMC-2, (**c**) nitrogen adsorption–desorption curve of NMC-2 adsorbent.

To investigate the structural composition of NMC-2, XRD analysis plots were performed. Figure 1a shows the XRD pattern of the NMC-2 composite before adsorption. The XRD pattern shows the corresponding strong and narrow peaks, from which it can be seen that the peaks of broad diffraction NMC-2 can correspond to the standard cards of Fe, C, Fe_7_C_3_, Fe_2_C, and FeC, indicating that the synthesized adsorbent is an iron-carbon composite. It can be indicated that mesoporous nitrogen-doped composites were formed during the carbonization process. During the experiments, it was found that the materials are magnetic, probably because of the presence of Fe, FeC, Fe_7_C_3_, Fe_2_C. Due to the magnetic properties of this type of material, rapid separation and recovery can be obtained under the conditions of an applied magnetic field, which allows easy separation of the adsorbent and metal ions from the wastewater^15^.

From the adsorption–desorption curves of adsorbent N_2_ in Fig. 1b, it can be seen that the NMC-2 isotherm belongs to the class IV curve, and the appearance of H3-type hysteresis loops is observed at the medium pressure end, and H3 is commonly found in aggregates with laminar structure, producing slit mesoporous or macroporous materials, which indicates that N_2_ condenses and accumulates in the pore channels, and these phenomena prove that NMC-2 is a porous material^16^. Figure 1c shows the pore size distribution of the adsorbent NMC-2 obtained according to the BJH calculation method, from which it can be seen that the pore size distribution is not uniform in the range, and most of them are concentrated below 20 nm, while according to Table 1, the specific surface area of the original sample of Fenton sludge and fly ash is 124.08 m^2^/g and 3.79 m^2^/g, respectively, and the specific surface area of NMC-2 is 228.65 m^2^/g. The Fenton The pore volume of the original samples of Fenton sludge and fly ash were 0.18 cm^3^/g and 0.006 cm^3^/g respectively, while the pore volume of NMC-2 was 0.24 cm^3^/g. The pore diameters of the original sample of Fenton sludge and fly ash were 5.72 nm and 6.70 nm respectively, while the pore diameter of NMC-2 was 4.22 nm. The above data indicated that the synthetic materials have increased the specific surface area and pore volume compared with the original samples, indicating that the doping of nitrogen can increase the specific surface area of the material. Since the pore size of mesoporous materials is 2–50 nm, NMC-2 is a porous material with main mesopores. Thanks to the large specific surface area provided by the mesopores, the material has a large number of active sites, and in addition, the mesopores can store more Cr(VI)^16^, which contributes to efficient removal.

**Table 1:** Total pore-specific surface area, pore volume, and pore size of BJH adsorption and accumulation of Fenton sludge, fly ash and NMC-2.

Name of material	Specific surface area (m^2^/g)	Pore volume (cm^3^/g)	Aperture (nm)
Fenton sludge	124.08	0.18	5.72
Fly ash	3.79	0.006	6.70
NMC-2	228.65	0.24	4.22

**Figure 2:**
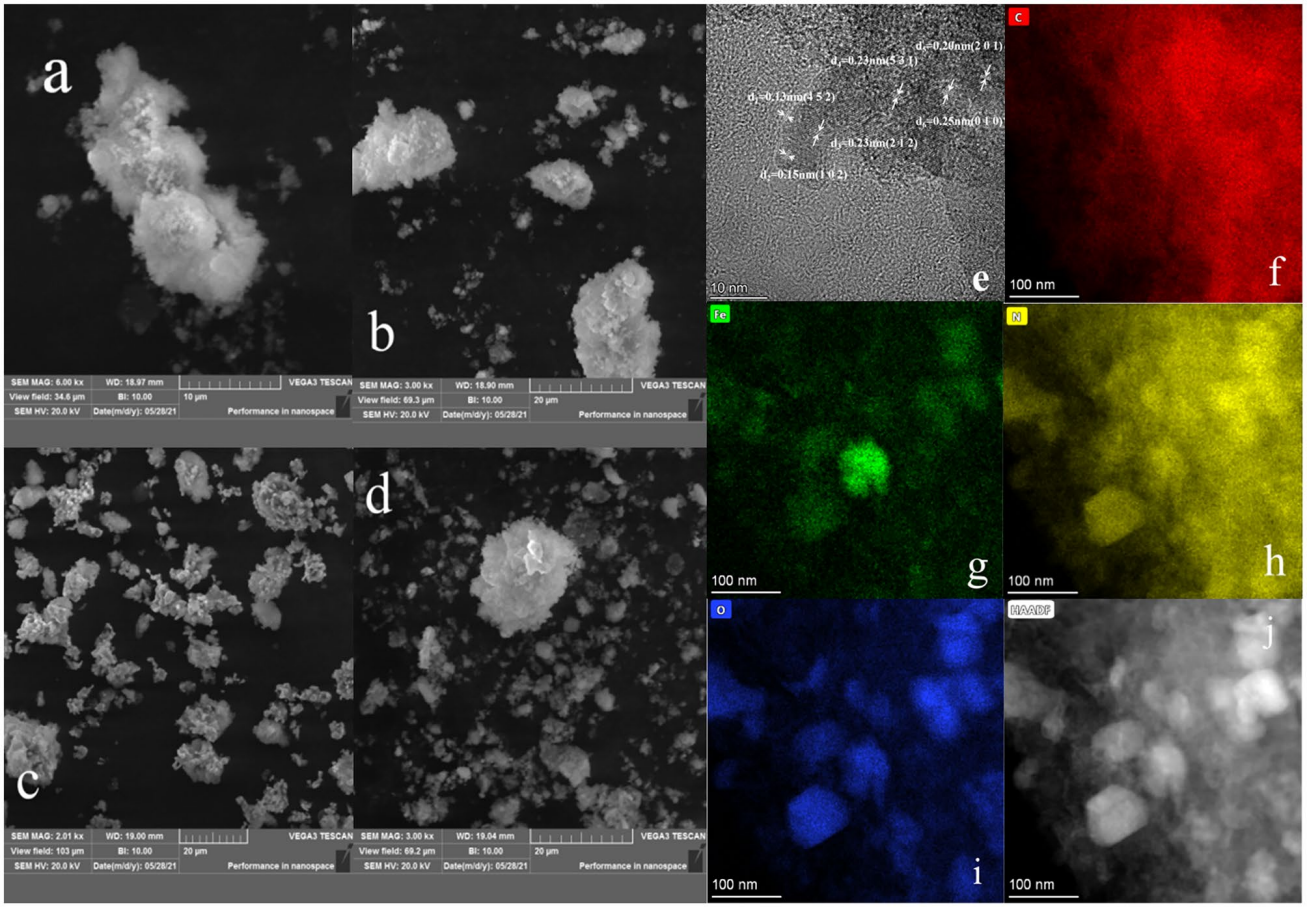
SEM, TEM and EDS testing of materials: (**a**–**d**) SEM image of NMC-2 adsorbent, (**e**) TEM image of NMC-2; (**g**–**i**) TEM-EDS spectrum of NMC-2, (**j**) TEM-EDS spectra of NMC-2 obtained from.

The morphological analysis of the material surface using SEM can see the surface structure and the pore structure of NMC-2. And Fig. 2a–d shows the swept electron microscope image of NMC-2. Figure 2a shows that the surface of the material is not smooth, and there are more lint-like fiber structures. The fibers in Fig. 2b are loosely and irregularly arranged, which may be due to the irregular morphology caused by the small particles of the NMC-2 sample. As shown in Fig. 2c and Fig. 2a there are more pores generated on the surface of NMC-2, which may be due to the addition of K_2_CO_3_ to urea and, Fenton sludge solution to generate CO_2_^17^.

These pores can provide many active sites, which is consistent with the results derived in Fig. 1, where NMC-2 is a mesoporous-dominated porous material, and also demonstrates that the addition of urea can provide a nitrogen source for the material, providing abundant active sites. Figure 2j depicts the TEM of NMC-2. the TEM images show that the synthesized NMC-2 has a folded structure with a surface covered by a carbon film, and the HRTEM (Fig. 2e) also confirms this result with a lattice spacing of 0.13, 0.15, 0.20, 0.23, 0.24, and 0.25 nm, corresponding to the (4 5 2) and (1 0 2) of C, the (2 0 1) of FeC) surface, the (2 1 0) surface of Fe_7_C_3_, the (5 3 1) surface of Fe_2_C, and the (2 0 1) surface of FeC, which also confirms the synthesis of the above substances. The corresponding EDS spectra of the dark field diagram NMC-2 were obtained from Fig. 2j, and the EDS spectra proved the presence of various elements: carbon (C) (Fig. 2f) from fly ash, iron (Fe) (Fig. 2g) from Fenton sludge, nitrogen (N) (Fig. 2h) from urea, and the presence of (O) (Fig. 2i), further confirming the successful preparation of NMC-2.

**Figure 3:**
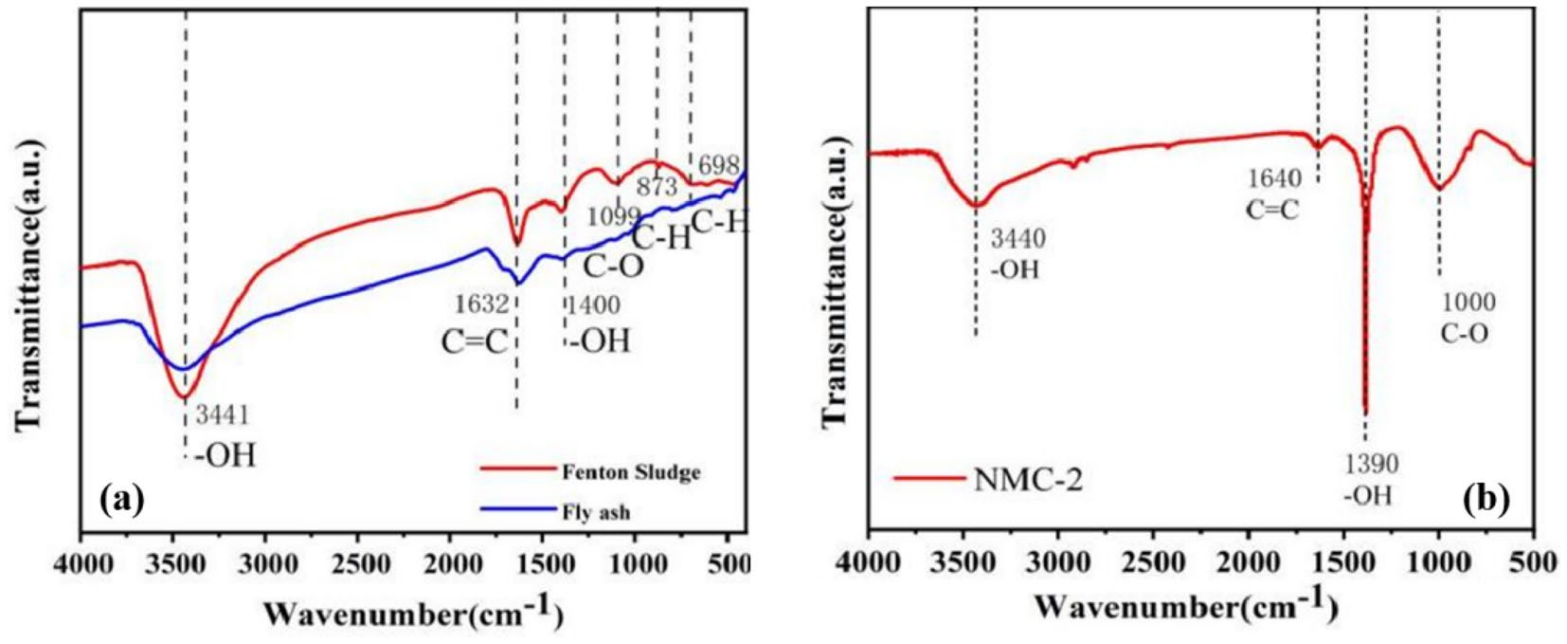
FTIR testing of materials: (**a**) FTIR image of Fenton sludge, fly ash, (**b**) Ftir image of NMC-2 adsorbent.

The type of functional groups and chemical bonding on the surface of the material can be analyzed by IR spectrogram analysis. Figure 3b shows the FTIR image of NMC-2 adsorbent 3440 cm^−1^ wide and strong absorption peak is due to the stretching vibration of –OH, there is a large amount of –OH present on the surface of the material; the peak appearing at 1640 cm^−1^ is –COOH. Characterization reveals that the –OH absorption peak is wider^18,19^. In addition, the absorptions at 1390 cm^−1^ and 1000 cm^−1^ were attributed to the bending of –OH vibrations of alcohols and phenol and the stretching vibration of C–O^20^. The above results indicate that the surface of NMC-2 contains a large number of oxygen-containing functional groups, and these functional groups can provide many active sites for the removal of Cr(VI). It was also found that the weak peaks corresponding to 573 cm^−1^ and 550 cm^−1^ were attributed to Fe–O groups^21^. The stretching of Fe–O may be due to the oxidation of loaded Fe^0^ and Fe^2+^ to Fe^3+^^22^. Figure 3a shows the Fenton sludge and fly ash FTIR images. It can be seen from the figure that the surfaces of Fenton sludge and fly ash contain a large number of oxygen-containing functional groups, the surface functional groups of the two raw materials are more abundant, and the functional groups of NMC-2 around 3441 cm^−1^, 1632 cm^−1^, and 1400 cm^−1^ are not significantly different from those of the raw materials, and the C–H stretching vibration peaks of NMC-2 around 873 cm^−1^ and 698 cm^−1^ is not obvious, which may be because the material the C–H bond on the surface of the raw material was oxidized to C–O in the process of synthesis.

### Cr(VI) adsorption experiment

#### Selection of adsorbent

**Figure 4:**
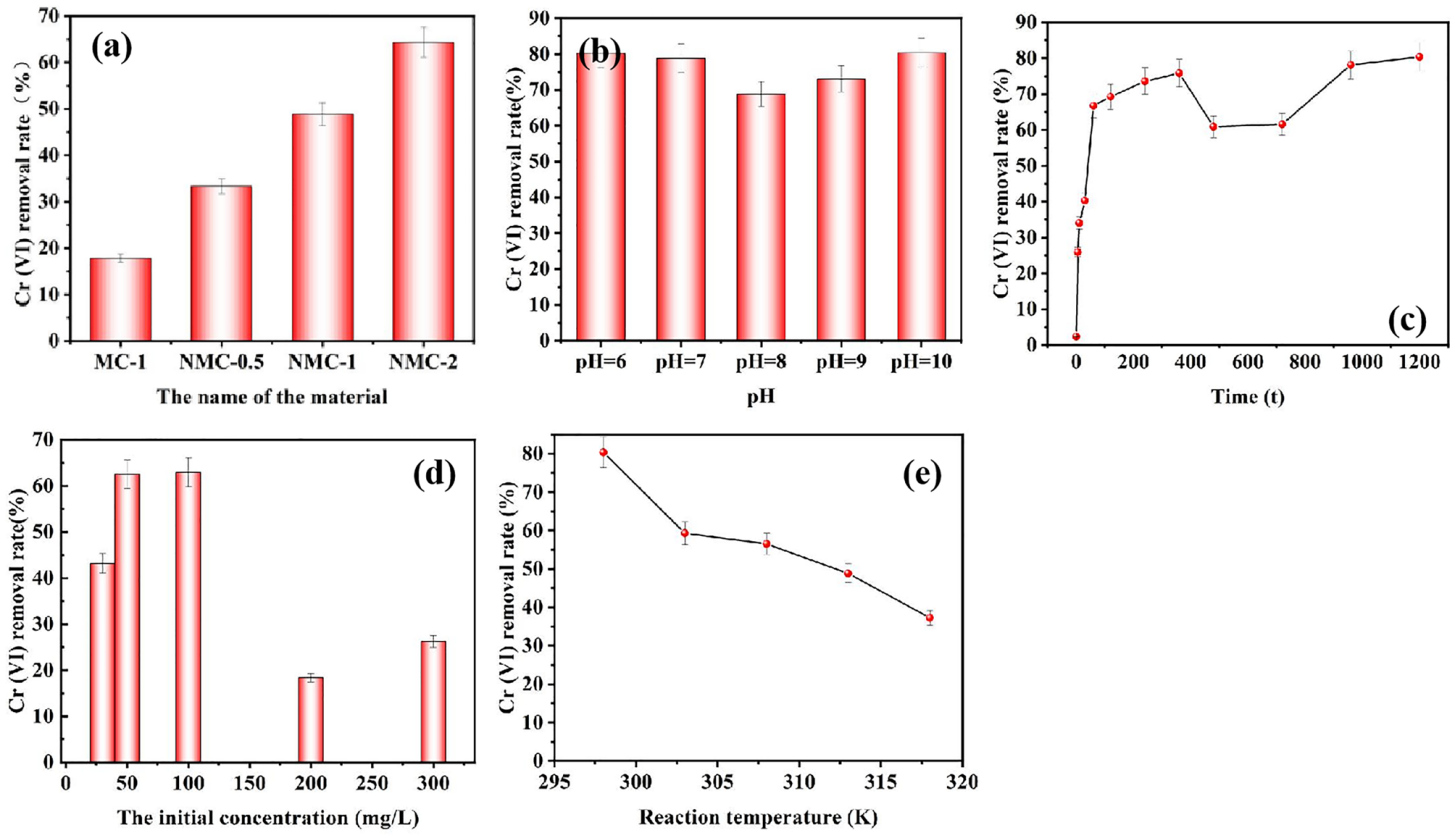
Adsorption influence factor test: (**a**) Effect of Fenton sludge and urea dosage on adsorption efficiency (m = 6 mg, C_0_ = 50 mg·L^−1^, V = 30 mL, T = 298 K, pH = 8, t = 20 h). (**b**) Effect of pH on Cr(VI) adsorption (m = 6 mg, C_0_ = 50 mg·L^−1^, V = 30 mL, T = 298 K, pH = 6–10, t = 20 h). (**c**) Effect of time on Cr(VI) adsorptions (m = 6 mg, C_0_ = 50 mg·L^−1^, V = 30 mL, T = 298 K, pH = 10, t = 0–1200 min). (**d**) Effect of concentration on Cr(VI) adsorption (m = 6 mg, C_0_ = 30–300 mg·L^−1^, V = 30 mL, T = 298 K, pH = 10, t = 20 h). (**e**) Effect of temperature on Cr(VI) adsorption (m = 6 mg, C_0_ = 50 mg·L^−1^, V = 30 mL, T = 298-318 K, pH = 10, t = 20 h).

To select the best adsorbent, Cr(VI) adsorption tests were performed on four adsorbents. Figure 4a shows the effect of Fenton sludge and the urea addition on the adsorption efficiency. The Cr(VI) removal rates of the four adsorbents were ranked from low to high: MC-1 < NMC-0.5 < NMC-1 < NMC-2. By comparing the Cr(VI) removal rates of MC-1 and NMC-1, it can be seen that the Cr(VI) removal rate of NMC-1 with 1 g of urea added was 48.8%. Cr(VI) removal rate of MC-1 with no urea was added, and its Cr(VI) removal rate was 17.8%, indicating that the addition of urea has a facilitating effect on the removal of Cr(VI). The addition of urea during the preparation of the adsorbent plays a role in providing a nitrogen source, indicating that doping with nitrogen can effectively improve the adsorption capacity of the material because the nitrogen-containing functional groups can provide more active sites and thus adsorb more Cr(VI). The comparison of the Cr(VI) removal rates of NMC-0.5, NMC-1, and NMC-2 showed that the Cr(VI) removal rate of Fenton sludge in NMC-0.5 was 33.3% with 0.5 g. The Cr(VI) removal rate of Fenton sludge in NMC-1 was 48.8% with 1 g. The Cr(VI) removal rate of Fenton sludge in NMC-2 was 64.3% with 2 g. It shows that the adsorption of material on Cr(VI) is related to the Fenton sludge dosage, and the higher the Fenton sludge dosage, the higher the adsorption efficiency. Meanwhile, in the adsorption of material with magnetite, it is found that NMC-2 has strong magnetism, and the adsorbent with stronger magnetism is easier to be magnetically separated and regenerated after adsorption, whether the adsorbent is easily separated and regenerated is very important in practical application, considering the magnetic separation performance and adsorption performance of the material, and choosing NMC-2 were selected for subsequent experiments considering the magnetic separation performance and adsorption performance of the material.

#### Effect of pH value

The pH is one of the factors that affect the performance of the adsorbent. The solution pH affects the protonation/deprotonation of the adsorbate and the surface charge of the adsorbent^4^. The effect of pH from 6.0 to 10.0 on the removal rate of Cr(VI) was investigated under the condition that other factors were kept constant. Figure 4b shows the test of the effect of pH on Cr(VI) adsorption, from the figure, it can be seen that NMC-2 has a higher Cr(VI) removal rate of 80.36% at pH = 10, and the Cr(VI) removal rate from pH = 6 to pH = 8 decreases continuously from the performance and the adsorption performance from pH = 8 to pH = 10 increases. Because pH determines the form of Cr(VI) in solution, Cr(VI) exists mainly in the form of CrO_4_^2−^ and HCrO_4_^−^ in aqueous solution, 1 < pH < 6.5, HCrO_4_^−^ is the main form; pH > 6.5, CrO_4_^2−^ is the main form. The decrease of removal rate from pH = 6 to pH = 8 is because the surface of NMC-2 is rich in oxygen-containing functional groups, which can form hydrogen bonds with HCrO_4_^−^, and it has lower adsorption free energy than CrO_4_^2−^, and is easily adsorbed; secondly, due to the acidic environment, the surface –COOH and -OH of NMC-2 can be protonated with H^+^, forming positively charged functional groups –OH^2+^, –COOH^2+^, which can bind the anions HCrO_4_^−^ and CrO_4_^2−^ through electrostatic interaction, resulting in higher adsorption of Cr(VI)^14,18,23,24^. When the pH was increased from 6 to 8, the alkalinity was strengthened and HCrO_4_^−^ was gradually converted to CrO_4_^2−^, which bound more oxygen-containing functional groups than HCrO_4_^−^ and reduced the adsorption, while OH– in the solution was easily bound to the acidic functional groups on the surface of NMC-2, which reduced the uptake of CrO_4_^2−^^23^. When pH = 8 to pH = 10 the removal rate of Cr(VI) gradually increased from 68.81 to 80.36%. Due to the large presence of CrO_4_^2−^ at increasing pH, at the same time a part of Cr(VI) was adsorbed and another part of Cr(VI) reacted with Fe^0^ and Fe^2+^ would form Cr(III) with a large amount of OH^−^, and Cr(III) and OH^−^ further formed precipitation. According to the above results, the optimal pH value for the experiment is pH = 10.

#### Effect of time

The effect of time from 0 to 1200 min on the removal rate of Cr(VI) was studied under the condition that other factors were kept constant. Figure 4c shows the test of the effect of time on the adsorption of Cr(VI). As can be seen from the figure, the reaction process of Cr(VI) on NMC-2 is divided into fast, diffusion, and equilibrium stages^25^. When 0–60 min, the adsorption is in the fast stage and the removal rate of Cr(VI) increases rapidly because the material has a porous structure, abundant surface functional groups, and active sites, which lead to the rapid adsorption of Cr(VI) by electrostatic force attraction. When 60–720 min, it belongs to the diffusion stage, the removal rate reaches 75.84% at 360 min, and then the removal rate slows down and decreases because the material surface functional groups and Cr(VI) are desorbed or the kinetics change leading to the reaction from fast to slow^26^. After that, the removal rate increased slowly and the active sites on the material surface were saturated until the equilibrium state was reached at 1200 min when the Cr(VI) removal rate could reach 80.36%.

#### Effect of initial concentration

The effect of concentration from 0 to 300 mg/L on the removal rate of Cr(VI) was studied under the condition that other factors were kept constant. The initial concentration also has an important effect on the adsorption, which not only affects the amount of Cr(VI) loaded on the NMC material, thus indirectly affecting the electron transport process, but also severely affects the distribution of heavy metal ions in the solution, and the removal rate of Cr(VI) can be maximized only under the optimum conditions^27^.

Figure 4d shows the effect of concentration on Cr(VI) adsorption, when the initial concentration increased from 30 to 100 mg/L, the removal rate of Cr(VI) gradually increased, probably because the initial concentration in the solution was low, the surface functional group of NMC-2 was more than Cr(VI) in solution, and Cr(VI) in solution was able to rapidly adsorb on NMC-2, resulting in a higher removal rate^28^. When the initial concentration increased from 100 to 200 mg/L, the Cr(VI) removal rate gradually decreased from 62.96 to 18.37%. It may be due to the increase in the initial concentration, the adsorption saturation of the functional groups on the surface of NMC-2 was reached in a short time, the adsorption rate slowed down, and the Cr(VI) removal rate decreased^17^. The results showed that the adsorption effect was better when the initial concentration was 100 mg/L.

#### Effect of temperature

Investigate the effect of temperature 273–315 K on the removal rate of Cr(VI) while keeping other factors constant. Figure 4e shows the effect of temperature on Cr(VI) adsorption, temperature is an important influencing factor, it will directly affect the removal rate of Cr(VI), as can be seen from the figure, when T = 318 K, the removal rate is only 37.26%, when T = 303 K, the removal rate is 59.30%, and the highest removal rate is 80.26% under the condition of T = 298 K. Therefore, the removal rate of Cr(VI) decreases with the increase in temperature, and the adsorption effect is better at T = 298 K.

#### Adsorption kinetics and isotherms

**Figure 5:**
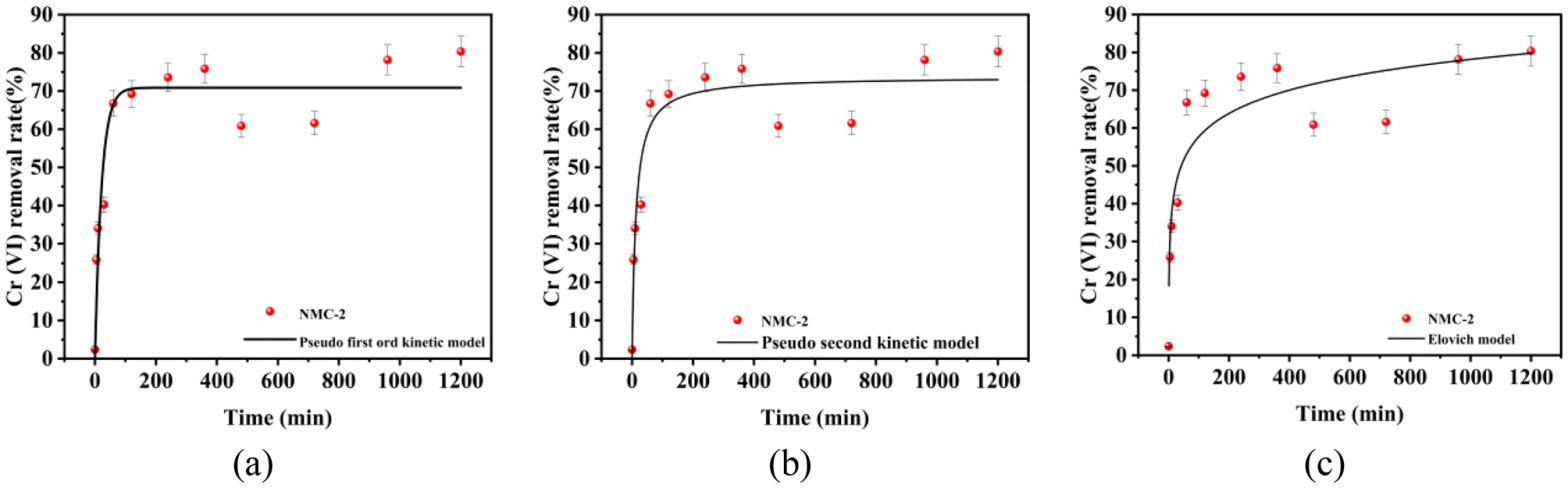
Adsorption influence factor test: (**a**) Pseudo first order dynamics model, (**b**) Pseudo second order dynamics model, (**c**) Elovich dynamics model.

The pseudo-primary kinetic model, pseudo second kinetic model, and modified Elovich model have fitted for the process of Cr(VI) removal by NMC-2, and the results are shown in Fig. 5a–c, and the correlation coefficients of the three kinetic models are shown in Table S2. The maximum adsorption amount of 70.87 mg/L was obtained, which was different from the experimental amount of 80.36 mg/L. This shows that the pseudo primary kinetic model is not suitable for describing the adsorption process of NMC-2 on Cr(VI).

The modified Elovich model was used to reflect the desorption process of non-uniform surface chemisorption. As shown in Table S2, the correlation coefficient *R*^2^ obtained by fitting the modified Elovich model was relatively small at 0.75593, and the maximum adsorption amount obtained by calculation was 58.58 mg/L, which differed significantly from the experimentally obtained adsorption amount of 80.36 mg/L. This shows that the modified Elovich model is not suitable to describe the adsorption process of NMC-2 on Cr(VI).

In addition Table S2 also calculates the relevant parameters of the pseudo second kinetic model, and the overall fitting order is: pseudo second kinetic model > pseudo-first-order kinetic model > Elovich model according to the decision coefficient *R*^2^. The pseudo second kinetic model gives the best fit (*R*^2^ > 0.9). It can be seen that the correlation coefficient *R*^2^ obtained by fitting the pseudo second kinetic model is 0.90658, which is closer to 1. Meanwhile, the maximum saturation adsorption amount calculated is 73.74 mg/L, respectively, which is less different from the experimentally obtained adsorption saturation adsorption value, and the pseudo second kinetic model has the best correlation with the NMC-2 removal of Cr(VI) system. It indicates that the kinetics of Cr(VI) adsorption by NMC-2 is more consistent with the pseudo second kinetic model. Therefore, it indicates that the kinetics of Cr(VI) adsorption by NMC-2 is more consistent with the pseudo second kinetic model, which further indicates that the process is a chemisorption process involving electron sharing or electron exchange, and the adsorption rate is controlled by chemisorption^29–31^. Also, this conclusion is in agreement with those obtained by other scholars using other adsorbents for Cr(VI) removal^19,21,32^. The adsorption reaction is a chemisorption accompanied by physical adsorption.

Table 2 compares the ability of the prepared NMC-2 composites with other materials for the removal of Cr(VI). The experimental adsorption amount of NMC-2, Q_max_ = 393.79 mg/g, was obtained by fitting the integral method, and the integral equation is shown below.

1 $${\text{Q}}_{{{\text{max}}}} = \int\limits_{0}^{{{\text{t}}_{{\text{e}}} }} {{\text{vdt}}}$$

where Q_max_, maximum adsorption capacity; *t*_*e*_, time to reach adsorption equilibrium, min; v, adsorbed solution volume, mL.

**Table 2:** Cr (VI) removal ability of NMC-2 composite compared with other material.

Adsorbents	Sorption capacity (mg/g)	References
Ppy/PANI/FBC	203.71	^21^
ALRCs/nZVI	196.5	^15^
FeOOH	30.3	^33^
Cd(II) MOF	228	^16^
Fe@PACB-700	22.24	^34^
N,S-C/Fe_3_O_4_-1	134	^35^
Nitrogen-containing Mesoporous carbon material-2 (this work)	393.79	

It was found through Table 2 that NMC-2 exhibited greater Cr(VI) adsorption capacity, indicating that the prepared composite can be used as a potential adsorbent for the effective cleaning of industrial wastewater with Cr(VI).

#### Adsorption thermodynamic

Adsorption isotherm refers to the relationship between adsorption capacity and gas phase pressure or concentration in the adsorption process under constant temperature. The most commonly used are Freundlich and Langmuir adsorption isotherms. Freundlich adsorption isotherm shows that the multilayer adsorption has a non-uniform distribution of functional groups, and the adsorbed molecules interact with each other. The formula is as shown in Equation S11. The Langmuir adsorption isotherm shows that the adsorbent forms a monomolecular layer on the surface of the adsorbent, each active site is the same and there is no interaction between adsorbed molecules, and the formula is shown in Equation S12^36^.

The equilibrium data are fitted by the Langmuir and Freundlich isotherm models, and the isotherm fitting curve is shown in Fig. S2, and it can be found that the Langmuir fitting curve *R*^2^ = 0.7978, the Freundlich fitting curve *R*^2^ = 0.9213, and the Freundlich *R*^2^ is closer to 1. It can be explained that the distribution of active sites on the surface of the adsorbent prepared in this study is not uniform, and there is a non-uniform surface condition on the surface in this experiment^30,37^. The adsorption process may be multilayer adsorption, and there are interactions between adsorbed Cr(VI) molecules. From Table S3, it can be seen that *n* =  − 1.45, 1/*n* =  − 0.689 < 1, and physical adsorption exists in the surface adsorption process^38^.

The adsorption thermodynamics can reflect the change of energy before and after adsorption, the adsorption thermodynamic parameters are as follows, in which the Gibbs free energy (ΔG), enthalpy (ΔH) entropy is calculated (ΔS), and the calculation equation is S13–S15. The fitting curve of lnK_d_ and 1/T is shown in Fig. S3. It can be seen that the slope after fitting is 8145.26, the intercept is − 24.55, and the fitting curve *R*^2^ = 0.90925, indicating a good linear correlation. The thermodynamic parameters obtained by calculation are shown in Table S4. It can be seen from the table that ΔG is negative, indicating that the adsorption process is spontaneous, at the same time, with the increase in temperature, the absolute value of ΔG decreases, indicating that low temperature is conducive to adsorption^39^. At the same time, the negative value of ΔH indicates that the adsorption process is an exothermic reaction, which is mainly monolayer adsorption accompanied by multilayer adsorption, which is consistent with the results of the thermodynamic model of adsorption. Generally, when ΔH is between 2.1 and 20.9 kJ/mol, which indicates that the adsorption is mainly physical; when ΔH is between 20.9 and 418.4 kJ/mol, it indicates that the adsorption is mainly chemical^40^. As can be seen from Table S4, the adsorption of NMC-2 is mainly chemical adsorption, which conforms to the fitting result of the kinetic model.

#### Reaction mechanism

**Figure 6:**
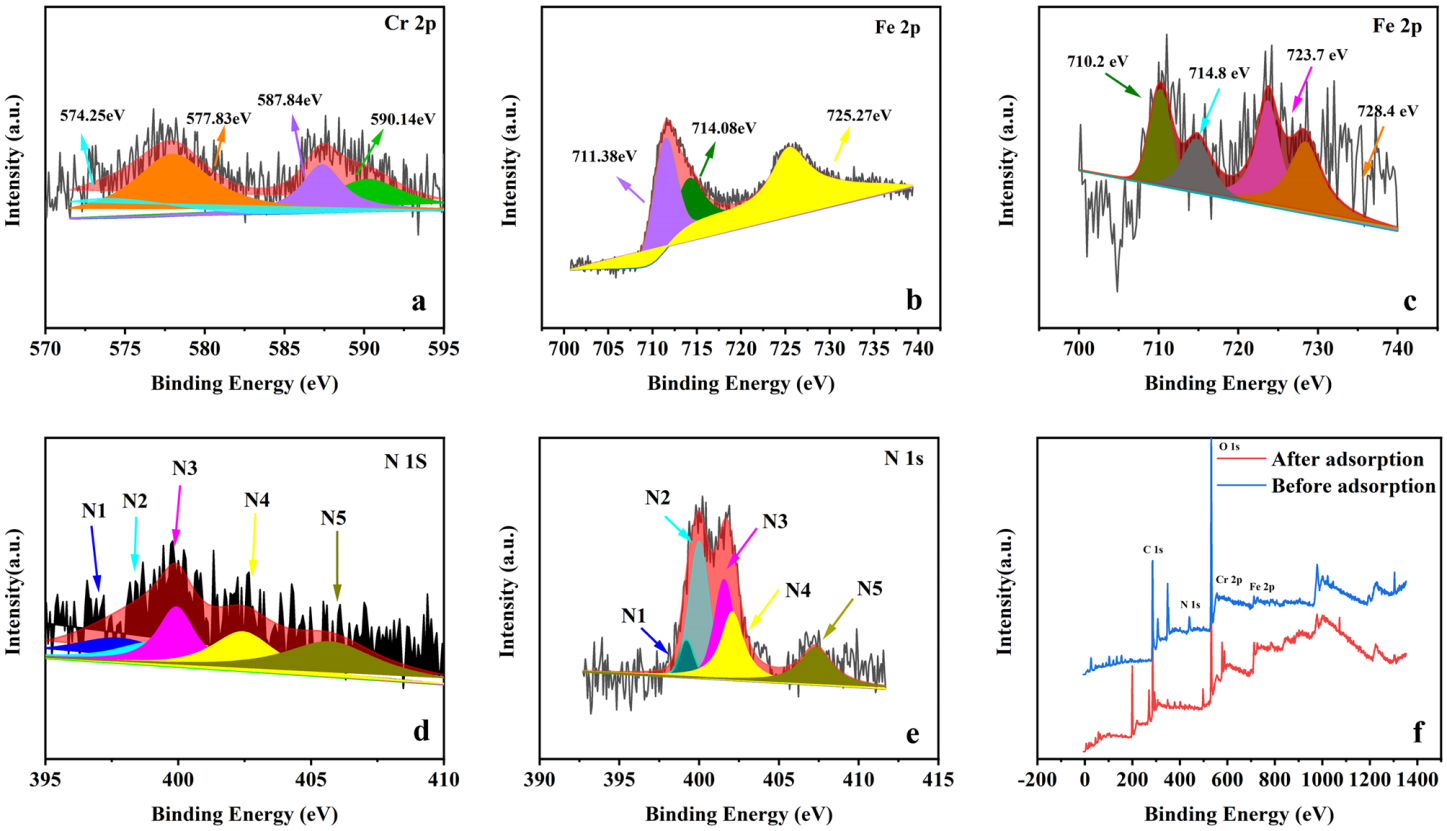
XPS testing of material: (**a**) XPS spectra of Cr 2P after adsorption of NMC-2, (**b**) XPS spectra of Fe 2P before adsorption of NMC-2, (**c**) XPS spectra of Fe 2P adsorbed by NMC-2, (**d**) XPS spectra of N 1s before adsorption of NMC-2, (**e**) XPS spectra of N 1s after adsorption of NMC-2, (**f**) full spectrum of NMC-2 before and after adsorption.

Figure 6c shows the XPS spectrum of Fe 2p after NMC-2 adsorption, XPS was used to further verify the valence state of Fe in the sample and the results show peak centers at 710.2 eV and 723.7 eV, indicating the presence of Fe^3+^ 2p3/2 and Fe^3+^ 2p1/2^33^. Also, the XPS analysis results revealed two additional peaks with higher energy at 714.8 eV and 728.4 eV as satellite peaks. It indicates the presence of trivalent iron oxides on the surface of NMC-2 after adsorption, which is consistent with the results obtained in XRD for the presence of FeC.

The reaction mechanism was determined by XPS measurements of the surface composition and material valence of NMC-2 before and after Cr(VI) adsorption. Figure 6f shows the full spectra of carbon, nitrogen, oxygen, and iron. The full spectrum fully demonstrates the entry of Fe and N elements into porous carbon during the carbon thermal process^41^. After adsorption, a peak of 578.08 eV appears, which is due to the presence of Cr 2p, which means that Cr(VI) is adsorbed by NMC-2, which also proves the XRD results in Fig. 1a. The Cr 2p peaks in Fig. 6a are mainly assigned to Cr(VI) 587.84 eV and 590.14 eV and Cr(III) 574.25 eV and 577.83 eV. The XPS results indicate that part of the highly toxic Cr(VI) will be adsorbed by NMC-2 and part of Cr(VI) will be reduced to Cr(III), which is further evidence that the reaction. This is further evidence that not only the adsorption reaction but also the redox reaction exists. Figure 6d shows the XPS spectra of N 1s before adsorption, which shows five fission peaks corresponding to N1, 397.77 eV (nitrogen bonding of metals), N2, 398.99 eV (pyridine-N), N3, 399.93 eV (pyrrole-N) and N4, 402.44 eV (graphite-N) and N5, 405.72 eV (nitrogen oxides), respectively^42,43^. The presence of the above five fission peaks after the adsorption of Cr(VI) can also be observed from the suction of Fig. 6e. A comparison of the results in Fig. 6d–e revealed that the peak area of N2 increased from 8.0 to 16.0%, N3 increased from 11.9 to 18.0%, N4 increased from 12.4 to 31.2%, the peak area content of N5 decreased from 64.8 to 31.9% after adsorption, and the above results indicated that pyridine-N, pyrrole-N, graphite-N, and nitrogen oxides provided the adsorption provided the driving force. Figure 6b shows the XPS spectrum of Fe 2p before adsorption, from which it can be found that before the adsorption of Cr(VI), Fe 2p was divided into three peaks: 711.38, 714.08, and 725.27 eV, which confirms the presence of Fe, Fe^2+^ and Fe^3+^^43^. After adsorption, Fe 2p peaks appeared at 710.2, 714.8, 723.7, and 728.4 eV, and no Fe, Fe^2+^, but Fe^3+^ were observed in the adsorbed peaks (Fig. 6f). This indicates that Fe, Fe^2+^, and Cr(VI) have undergone redox reactions to form Fe^3+^. To further illustrate the reaction mechanism, the Cr(III) and Cr(VI) ratios were calculated using XPS data as shown in Table S5. The results showed that Cr(III) and Cr(VI) accounted for 60.65% and 39.35% of Cr, respectively, indicating the presence of oxidation behavior during the reaction and the predominance of redox reaction^43,44^.

**Figure 7:**
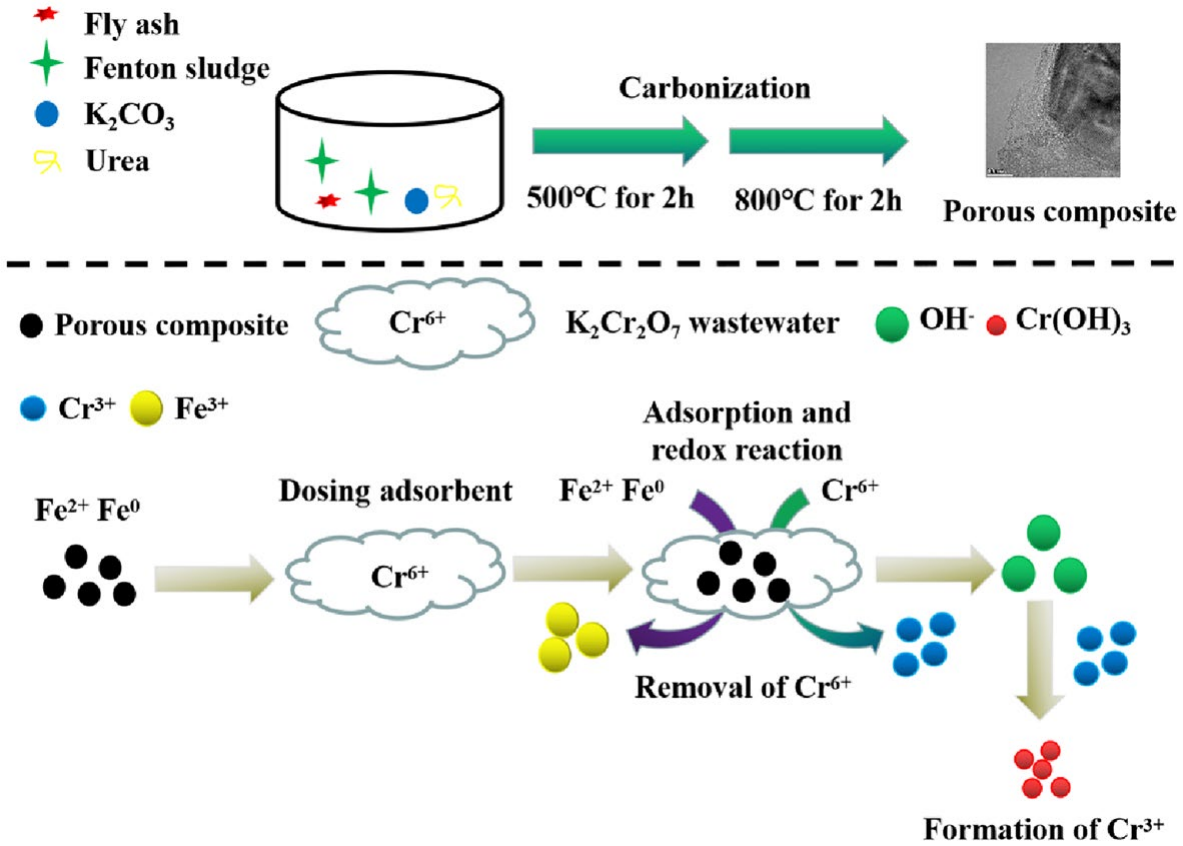
Reaction mechanism diagram.

Based on the above analysis, the reaction mechanism of Cr(VI) is summarized as follows: firstly, a part of Cr(VI) is adsorbed by the negative charge on the surface of NMC-2 due to electrostatic gravitational force. Meanwhile, the surface of NMC-2 is rich in functional groups and has a large specific surface area, which is beneficial to enhance the removal rate of Cr(VI)^43,45,46^. Secondly, Fe and Fe^2+^ transfer electrons and react with the redox reaction of Cr(VI) through the porous channels on the surface of NMC-2 (Equations 2-3). Finally, as the Cr(VI) reaction generates a large amount of OH^−^, resulting in Cr(III) can be present in the form of a precipitate. The reaction mechanism is shown in Fig. 7.

2 $${\text{Fe}}^{0} + {\text{CrO}}_{4}^{2 - } + 4{\text{H}}_{2} {\text{O}} = {\text{Cr}}^{3 + } + {\text{Fe}}^{3 + } + 8{\text{OH}}^{ - }$$

3 $$3{\text{Fe}}^{2 + } + {\text{CrO}}_{4}^{2 - } + 4{\text{OH}}^{ - } + 4{\text{H}}_{2} {\text{O}} = {\text{Cr}}({\text{OH}})_{3} + 3{\text{Fe}}({\text{OH}})_{3}$$

## Discussion

NMC-2 is an environmentally friendly adsorbent synthesized using iron-based solid waste Fenton sludge and fly ash for the removal of Cr(VI) from high pH industrial chromium-containing wastewater. The synthesis method is simple, easy to operate, and low-cost. The adsorbent was found to be predominantly mesoporous, with a large number of oxygen-containing functional groups formed on the surface and possessing a large number of active sites. The adsorption kinetics followed a pseudo second kinetic model with a maximum adsorption capacity of about 393.79 mg/g. The process is a chemisorption process involving electron sharing or electron exchange, and the adsorption rate is controlled by chemisorption. The reaction mechanism is a portion of Cr(VI) is adsorbed by the negative charge on the surface of NMC-2 due to electrostatic gravitational force. At the same time, the material surface is rich in functional groups and has a large specific surface area, which can remove Cr(VI) efficiently. Furthermore, Fe and Fe^2+^ transfer electrons and react with the redox reaction of Cr(VI) through the porous channels on the surface of NMC-2. Finally, as the Cr(VI) reaction generates a large amount of OH-, resulting in Cr(III) can be present in the form of precipitation. The prepared materials have both the adsorption properties of conventional adsorbents and strong magnetic properties compared to conventional mesoporous nitrogen-doped composites. The magnetic properties of the material allow rapid separation and recovery under the condition of an applied magnetic field, thus making it easy to separate the adsorbent and metal ions from the wastewater. Meanwhile, this experiment has achieved a good adsorption effect in the treatment of chromium-containing wastewater on the one hand, and the cost of preparation is relatively lower on the other hand, and it can also achieve the purpose of treating waste with waste.

## Methods

### Materials and equipment

Fenton sludge was obtained from Daitansha Wastewater Treatment Plant, in Guangzhou, China. Fly ash was obtained from Xinjiang Thermal Power Plant, Xinjiang, China. The elemental content analysis of Fenton sludge is shown in Table S6, the elemental analysis of fly ash is shown in Table S7, and the organic elemental analysis test of fly ash is shown in Table S8. The other reagents used in the experiments were obtained from Tianjin Sailing Chemical Reagent Technology Co. All reagents were analytically pure, and all reagents were not further processed. The water used in the experiments was ultrapure.

### Preparation method of NMC

The preparation process of adsorbent was as follows: Fenton sludge and fly ash were dried and ground, passed through a 200 mesh sieve in a three-mouth flask, and a certain amount of urea and potassium carbonate was added, where the mass, mass ratio, and sample name of Fenton sludge, fly ash, and urea was added under the same conditions as shown in Table S1, and then 30 mL of ultrapure water was added and ultrasonically dispersed for 30 min at room temperature. The samples were then ground and passed through a 200 mesh sieve, and the sieved samples were placed in a tube furnace under the condition of N_2_ and a heating rate of 5 °C/min, and then heated to 500 °C for 2 h, and then heated to 800 °C for 2 h, and then washed with water by centrifugation to neutral, and then washed with ethanol by centrifugation for 2–3 times to obtain the prepared samples. The prepared mesoporous nitrogen-doped carbon material was obtained by natural drying, named Nitrogen-containing Mesoporous Carbon material-n (NMC-n), where n is the mass of the added Fenton sludge. According to the above method, the magnetic porous carbon material prepared without the addition of urea is named Mesoporous Carbon material (MC-n), where n is the mass of the added Fenton sludge.

### Selection of adsorbent

The Cr(VI) solution prepared by K_2_Cr_2_O_7_ was adjusted to pH = 8 with 1 mol/L HCl and 1 mol/L NaOH solution using a pH meter. And pH = 8 was taken as the pH range of the probe was pH = 6–10. Four samples (6 mg each) of MC-1, NMC-0.5, NMC-1, and NMC-2 were added into brown vials with 30 mL of Cr(VI) solution prepared with 50 mg/L K_2_Cr_2_O_7_ at pH = 8 and T = 298 K and shaken at 250 rpm for 20 h. After the adsorption, the supernatant was filtered, and the separated solids were washed and stored for regeneration. The concentration of Cr(VI) was measured by UV spectrophotometer and the material with a better adsorption effect was selected.

### Adsorption experiment of Cr (VI)

Using the adsorbent prepared above for the adsorption test, 0.5 g of K_2_Cr_2_O_7_ solid powder was taken and dried at 105 °C for 2–3 h, naturally cooled, and weighed 0.2829 g in a beaker, dissolved with a small amount of water, quantitatively transferred to a 1000 mL brown volumetric flask, diluted with water to the scale, shaken well, labeled and set aside. Take 150 mL of the solution in 5 portions and adjust the pH of the above solution to 6, 7, 8, 9, and 10 respectively with 1 mol/L HCl and 1 mol/L NaOH using a pH agent. After adsorption, the suspension was separated by an external magnet and the supernatant was filtered by an injection membrane with a pore size of 0.45 μm. The separated solid was washed and stored for regeneration, and the collected supernatant was analyzed by UV spectrophotometry. The collected supernatant was analyzed by UV spectrophotometric method. The removal rate of Cr(VI) was calculated using Equations S1–S4. The absorbance was measured at 540 nm with a spectrophotometer (752/752 N, China) and a 10 mm glass cuvette at a UV spectrophotometer. The adsorption capacity (q-value) is calculated as shown in Equation S1.

The main spectrophotometer used in this study was the Chinese national standard GB/7467-87 “Water quality-Determination of hexavalent chromium-Diphenylcarbodihydrazide spectrophotometric method”. The experimental instrument was calibrated before use, and the standard curve is shown in Fig. S2, and the standard curve is shown in Equation S16.

### Effect of pH value

The Cr(VI) solution prepared by K_2_Cr_2_O_7_ was adjusted to the pH of the solution with 1 mol/L HCl and 1 mol/L NaOH solution using a pH meter. 30 mL of 50 mg/L Cr(VI) solution and 6 mg of NMC-2 were added to a brown vial and shaken at 250 rpm at T = 298 K under the above pH conditions. After adsorption, the supernatant was filtered, and the separated solid was washed and stored for regeneration. The concentration of Cr(VI) was measured by a UV spectrophotometer to investigate the effect of pH on the adsorption of Cr(VI).

### Effect of time

The Cr(VI) solution prepared from K_2_Cr_2_O_7_ was adjusted to pH = 10 with 1 mol/L HCl and 1 mol/L NaOH solution using a pH meter. 30 mL of 100 mg/L Cr(VI) solution and 6 mg NMC-2 were added to a brown vial for the specified periods t = 0, 5, 10, 30, 60, 120, 240, 360, 480, 720, 960, and 1200 min. The adsorption was carried out by shaking at t = 298 K, pH = 10, and 250 rpm on a shaker. After adsorption, the supernatant was filtered, and the separated solid was washed and stored for regeneration. The concentration of Cr(VI) was measured by a UV spectrophotometer to investigate the effect of time value Cr(VI) adsorption.

### Effect of initial concentration

The Cr(VI) solution prepared by K_2_Cr_2_O_7_ was adjusted to pH = 10 with 1 mol/L HCl and 1 mol/L NaOH solution using a pH meter. 30 mL of 30, 50, 100, 200, and 300 mg/L of Cr(VI) solution and 6 mg of NMC-2 were added to a brown vial at T = 298 K, pH = 10, and shaking at 250 rpm for 1200 min.

### Effect of temperature

The Cr(VI) solution prepared by K_2_Cr_2_O_7_ was adjusted to pH = 10 with 1 mol/L HCl and 1 mol/L NaOH solution using a pH meter. 30 mL of 100 mg/L Cr(VI) solution and 6 mg NMC-2 were added to a brown vial and adsorbed at T = 298, 303, 308 K, pH = 10, shaking at 250 rpm for 1200 min. After adsorption, the supernatant was taken and filtered, and the separated solid was washed and stored for regeneration. The concentration of Cr(VI) was determined by a UV spectrophotometer. The concentration of Cr(VI)was measured by a UV spectrophotometer to investigate the effect of temperature on the adsorption of Cr(VI).

### Characterization method

X-ray fluorescence spectrometer (XRF, PANalytical Axios) is used to determine the chemical composition and elemental content. X-ray diffractometer (XRD, Burker Advance D8) is used for the analysis of the crystal structure, the scanning angle is 5°–90°, and the continuous scanning rate is 10°/min, using copper target card radiation for the working wavelength Scanning electron microscopy (SEM, NovaNanoSEM50) was used for grain size and morphology analysis. X-ray photoelectron spectrometer (XPS, PHI5000Versaprobe-II) is used to analyze the valence state of the material before and after adsorption, to assist in understanding the state, structure, and properties of the material. Power: 50 W; Voltage: 15 kV; Anode; Al target; Calibration: C1S (284.8 eV); Pass energy: 46.95 eV. N_2_ physical adsorption/desorption instrument (Tristar II 3020) is used to understand the specific surface area, pore size range, average pore size, and pore capacity of materials. The adsorbent was first excluded from the air at 300 °C for 3 h. Nitrogen was used as the adsorbent and helium as the carrier gas, and the tests were performed in a liquid nitrogen environment (− 196 °C). Fourier transform infrared spectroscopy (FTIR, Nicolet IS50) was used to analyze the changes in surface functional groups before and after adsorption. FEI Tecnai G2 F2 emission lens electron microscopy (TEM, EDS, OXFXRD X-max 80 T) was used to analyze the surface microstructure and elements of the adsorbent. Samples are sonicated in ethanol liquid, taken and added to a copper grid covered with carbon film, dried, and observed. H2-TEM allows observation of the surface morphology of the adsorbent, and EDS is used for elemental analysis. Organic elemental analyzer (Elementary: Vario EL cube) with test mode CHNS mode, analyzes the content of elemental carbon in the fly ash to provide a basis for its preparation of materials with carbon source.

## Supplementary Information

Supplementary Information.

## Data Availability

The data that support the findings of this study are available from the corresponding author on request.
