# Supplementary material for: Study on adsorption of hexavalent chromium by composite material prepared from iron-based solid wastes
Source: Sci Rep. 2023 Jan 4;13:135. doi: 10.1038/s41598-023-27414-9 (PMC9813190; doi:10.1038/s41598-023-27414-9)
Supplement: Supplementary file 1 — Supplementary Information. [file 41598_2023_27414_MOESM1_ESM.docx]

**Supporting Information**

**Study on adsorption of hexavalent chromium by composite material prepared from iron-based solid wastes**

Jiamin Qi ^a^, Bin Li ^a,b^,*^*^*, Pengxiang Zhou ^a^, Xintai Su ^c^,*^*^*, Di Yang ^a^, Jinxiong Wu ^d^, Zixuan Wang^a^, Xiangjing Liang ^e^

*^a^ Faculty of Environmental Science and Engineering, Kunming University of Science and Technology, Kunming, 650500, China*

*^b^ National-Regional Engineering Center for Recovery of Waste Gases from Metallurgical and Chemical Industries, Kunming, 650500, China*

*^c^ School of Environment and Energy, Guangdong Provincial Key Laboratory of Solid Wastes Pollution Control and Recycling, South China University of Technology, Guangzhou, Guangdong, 510006, China*

*^d^ Yili Normal University. Yining, Xinjiang, 835000, China*

*^e^Guangzhou Haitao Environmental Protection Technology Company Limited, Guangzhou, Guangdong, 510006, China*

**Analyses of the removal rate of Cr(Ⅵ) data**

The removal rate of Cr(Ⅵ) was calculated according to the following equation [1]:

$R_{t}\text{(\%)}=\frac{(C_{0}-C_{1})}{C_{0}}\times\text{100\% (S1)}$

$\text{ }q_{t}=\frac{V\cdot(C_{0}-C_{1})}{m}\text{ (S2)}$

$R_{t}\text{(\%)}=\frac{(C_{0}-C_{e})}{C_{0}}\times\text{100\% (S3)}$

$\text{ }q_{e}=\frac{V\cdot(C_{0}-C_{e})}{m}\text{ (S4)}$

Where:

*R_t_* (%)—Cr(Ⅵ) removal rate at time t,%;

*R_e_*(%)—Cr(Ⅵ) removal rate at equilibrium,%;

*q_t_*—adsorption capacity of Cr(Ⅵ) at time t, mg/g;

*q_e_*—Cr(Ⅵ) adsorption capacity at equilibrium, mg/g;

*C_0_*—concentration of Cr(Ⅵ) in solution before adsorption, mg/L;

*C_t_*—time of adsorption t, the concentration of Cr(Ⅵ) in solution, mg/L;

*C_e_*—Cr(Ⅵ) concentration in solution after adsorption (concentration at equilibrium), mg/L;

*V*—solution volume (adsorbate volume), L;

*m*—the quality of adsorbent applied, g;

**Adsorption Kinetic**

The adsorption rate is determined by the diffusion rate of the adsorbed species at different reaction stages in the reaction phase. Adsorption kinetics is mainly used to describe the adsorption rate of the adsorbent to the solvent, and the adsorption rate controls the residence time of the adsorbate on the solid-liquid surface. To explore the adsorption characteristics of Cr(Ⅵ), the Pseudo first order dynamics model, the Pseudo second order kinetic model, and the modified Elovich model were used to fit the experimental data. The fitting reference equations are as follows[2].

**Pseudo first order dynamics model**

$$\frac{d_{qe}}{d_{t}}\text{ }=R_{1}\text{(}q_{e}-q_{t}\text{) (S5)}$$

Where:

*t*—adsorption time, min;

*q_e_*—adsorption capacity at equilibrium, mg/g;

*q_t_*—adsorption capacity at time t, mg/g;

*k_1_*—the adsorption rate constant of Pseudo first order dynamics model, min^-1^;

From the boundary condition of the differential equation *t* = 0, *q_t_* = 0, the above equation can be integrated to obtain [3]:

$$\text{In(}q_{e}-q_{t}\text{) }=\text{In}q_{e}\text{-}\frac{k_{1}}{2.303}t\text{ (S6) }$$

If a straight line can be obtained by plotting$\text{In(}q_{e}-q_{t}\text{) }$against *t*, it shows that the adsorption mechanism conforms to the Pseudo first order dynamics model. The adsorption capacity at adsorption equilibrium was obtained through experiments.

**Pseudo second order dynamics model**

A Pseudo second order dynamics model can be described by [4]:

$$\frac{d_{qe}}{d_{t}}\text{ }=k_{2}\text{(}q_{e}-q_{t}\text{)}^{2}\text{ (S7)}$$

Where:

*t*—adsorption time, min;

*q_e_* — adsorption capacity at equilibrium, mg/g;

*q_t_*—adsorption capacity at time t, mg/g;

*k_2_*—adsorption rate constant of Pseudo second order dynamics model, g·mg^-1^·min^-1^.

Integrating the above equation yields:

$$\frac{t}{q_{t}}=\frac{1}{k_{2}q_{e}^{2}}+\frac{t}{q_{e}} \text{ (S8)}$$

The time *t* was plotted $\frac{t}{q_{t}}$, and a straight line was obtained by linear fitting of the data. The equilibrium adsorption capacity and Pseudo second order rate constant *K_2_* were calculated by the slope and intercept of the straight line.

**Elovich model**

An Elovich model can be described by [5]:

$$\frac{d_{q_{t}}}{d_{t}}\text{ }=\alpha e^{-\beta q_{t}}\text{ (S9)}$$

Where:

α is the initial adsorption rate constant;

$$q_{t}=\frac{1}{\beta}\text{ln}\left(1+\text{K}\beta\text{t}\right)\text{ (S10)}$$

The parameter *β* is related to the surface coverage and activation energy of the chemisorption [6].

**Freundlich adsorption isotherm model**

The Freundlich isotherm is as follows [7]:

$$q_{e}=K_{f}C_{e}^{1/n} (S11)$$

Where:

C_e_—equilibrium concentration, mg/L;

*q*_e_—adsorption capacity at equilibrium, mg/g;

*q_max_*—maximum adsorption capacity, mg/L;

*K_f_*—Freundlich constant, mg^1−1/n^ L^1/n^ g^−1^;

*n*— the adsorption intensity.

**Langmuir adsorption isotherm model**

The Langmuir isotherm is as follows[8]:

$$q_{e}=\frac{q_{max}K_{L}c_{e}}{1+K_{L}C_{e}} (S12)$$

Where:

C_e_—equilibrium concentration, mg/L;

*q*_e_—adsorption capacity at equilibrium, mg/g;

*q_max_*—maximum adsorption capacity, mg/L;

*K_L_*—Langmuir constant, (L/mg).

**Calculation formula of thermodynamic parameter**

$\ln K_{d}=-\frac{\Delta H}{RT}+\frac{\Delta S}{R}$ (S13)

$$\Delta G=\Delta H-T\Delta S (S14)$$

$$K_{d}=\frac{q_{e}}{\mathrm{Ce}} (S15)$$

Where:

ΔG— Gibbs free energy, kJ/mol;

ΔH—enthalpy, kJ/mol;

ΔS—entropy, J/mol·K;

C_e_—equilibrium concentration, mg/L;

*q*_e_—adsorption capacity at equilibrium, mg/g.

ΔH and ΔS are obtained from the slope and intercept of the plot of lnK_d_ versus 1/T[9].

**Table S1. Proportion and name of sampled**

| **Abbreviation** | **Proportion**  **sample name** | **Fenton Sludge(g)** | **Fly ash(g)** | **Urea(g)** | **Potassium Carbonate(g)** |
| --- | --- | --- | --- | --- | --- |
| NMC-1 | 1：1：1：1 | 1 | 1 | 1 | 1 |
| MC -1 | 1：1：0：1 | 1 | 1 | 0 | 1 |
| NMC-0.5 | 1：2：2：2 | 0.5 | 1 | 1 | 1 |
| NMC-2 | 2：1：1：1 | 2 | 1 | 1 | 1 |


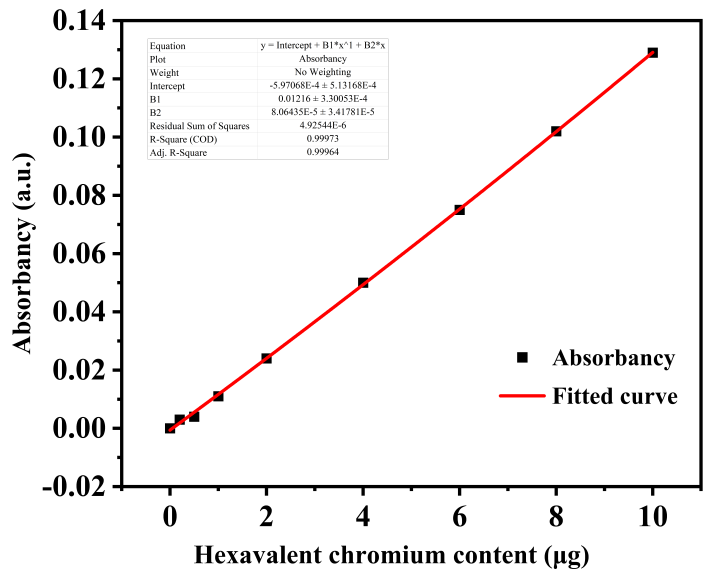


**Fig. S1.** UV spectrophotometric standard curve

Standard curve equation:

$x=(y+0.0013)/$0.0129 (S16)

**Table S2.** Kinetics related parameter

| **Adsorbent** | **Pseudo first order dynamics model** | | | **Pseudo second order dynamics model** | | | **Elovich model** | | |
| --- | --- | --- | --- | --- | --- | --- | --- | --- | --- |
|  | *K_1_* | *q_e_*/mg·g^-1^ | *R^2^* | *K_2_* | *q_e_*/mg·g^-1^ | *R^2^* | *β* | *K_3_* | *R^2^* |
| **NMC-2** | 0.04903 | 346.3900 | 0.77812 | 0.000236 | 361.1076 | 0.84115 | 69.39253 | 0.112520 | 0.73238 |


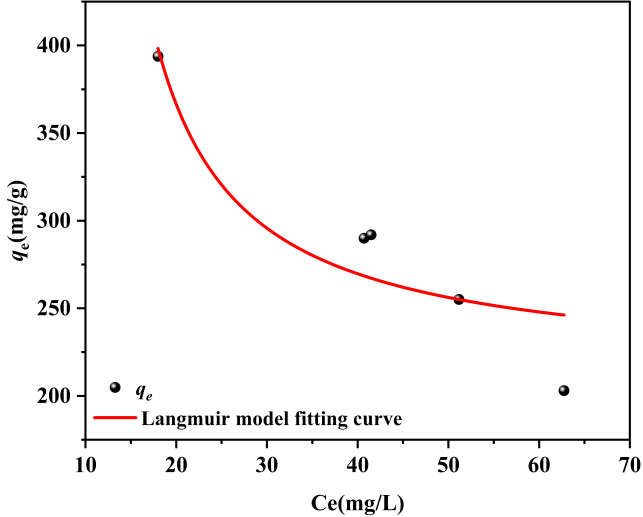

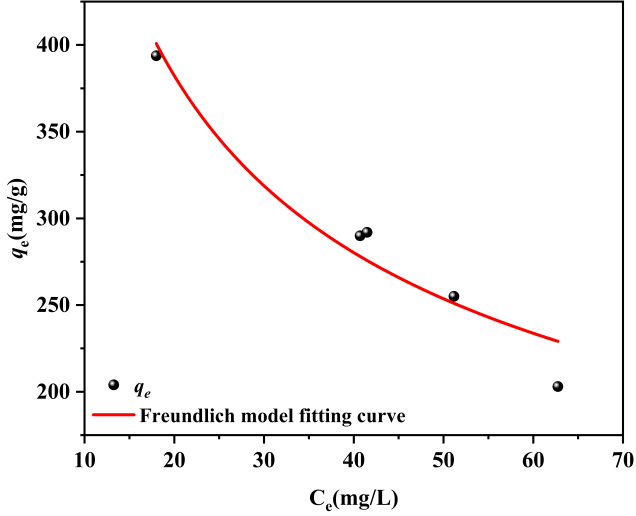


**Fig. S2.** Thermodynamic fitting curve

1. Freundlich model fitting curve; (b) Langermuir model fitting curve

**Table S3.** Thermodynamic Parameters

| Langmuir | | | Freundlich | | |
| --- | --- | --- | --- | --- | --- |
| *q_max_* (mg/g) | *K_L_* | *R*^2^ | *K_f_* (mg/g) | *n* | *R^2^* |
| 213.37 | -8.36 | 0.7978 | 383.83 | -1.45 | 0.9213 |


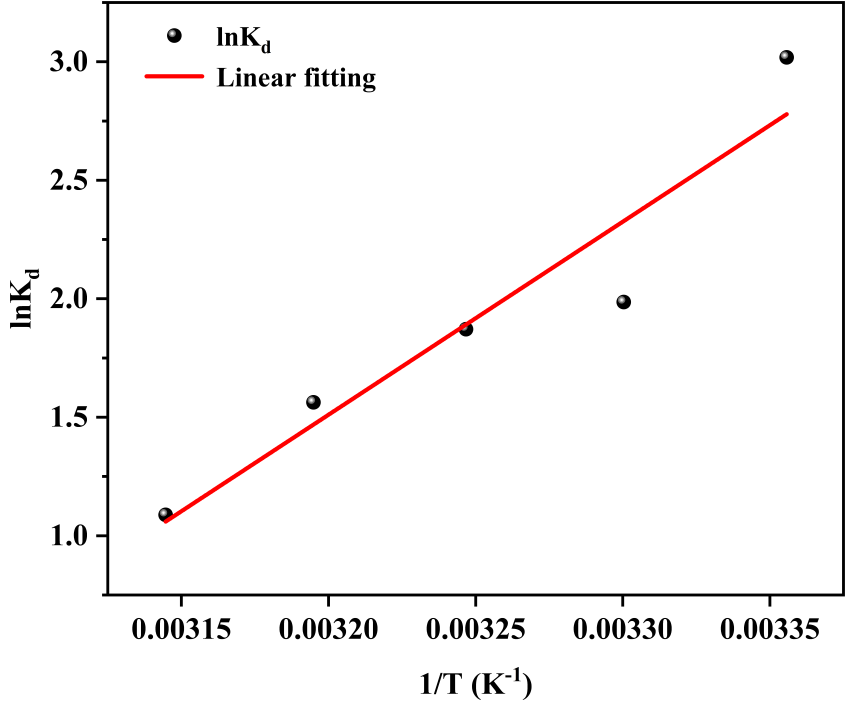


**Fig. S3**  Plots of lnK_d_ versus 1/T

Standard curve equation:

$$y=-24.55x+8145.26 (S17)$$

**Table S4.** Thermodynamic parameters for an adsorption Cr(VI)

| T (K) | ∆H (kJ/mol) | ∆S (kJ/mol·K) | ∆G (kJ/mol) |
| --- | --- | --- | --- |
| 298 | -24.55 | 8.145 | -7.48 |
| 303 |  |  | -5.00 |
| 308 |  |  | -4.79 |
| 313 |  |  | -4.08 |
| 318 |  |  | -2.88 |

**Table S5.** Peak area ratio of N element and Cr element

| **Peak area ratio**  **Element** | **N1** | **N2** | **N3** | **N4** | **N5** | **Cr^6+^** | **Cr^3+^** |
| --- | --- | --- | --- | --- | --- | --- | --- |
| The area ratio of the pre-adsorption peak | 3.0% | 8.0% | 11.9% | 12.4% | 64.8% | —— | —— |
| The ratio of peak area after adsorption | 3.0% | 16.0% | 18.0% | 31.2% | 31.9% | 39.4% | 60.7% |

**Table S6.** Elemental composition and percentage content in Fenton sludge samples

| **Element** | **Percent content%** | **Element** | **Percent content%** | **Element** | **Percent content%** |
| --- | --- | --- | --- | --- | --- |
| Al | 0.3419 | K | 0.8639 | Cr | 0.0148 |
| Na | 0.9975 | Ti | 0.8043 | Cl | 0.6102 |
| Mg | 1.0116 | Fe | 54.7715 | Ca | 5.5474 |
| Si | 0.9387 | O | 27.0222<balance> | S | 1.5229 |
| P | 0.4229 | Zn | 0.0898 | Sr | 0.0282 |
| Mn | 0.2280 |  |  |  |  |

**Table S7.** Elemental composition and percentage content in fly ash samples

| **Element** | **Percent content%** | **Element** | **Percent content%** | **Element** | **Percent content%** |
| --- | --- | --- | --- | --- | --- |
| Al | 2.0313 | K | 0.1706 | Pb | 0.0098 |
| Na | 0.2773 | Ti | 0.2554 | Cl | 0.2954 |
| Mg | 0.3475 | Fe | 3.7205 | Ca | 4.2770 |
| Si | 2.2861 | O | 14.3415<balance> | S | 0.5532 |
| P | 0.0063 | Zn | 0.0055 | Pb | 0.0098 |
| Cr | 0.0059 | Rb | 0.0037 | Cl | 0.2954 |
| Mn | 0.0133 | Ba | 0.0253 | Ca | 4.2770 |

**Table S8.** Organic element analysis test result

| N(%) | C(%) | H(%) | S(%) | O(%) |
| --- | --- | --- | --- | --- |
| 0.9 | 48.09 | 3.233 | 0.424 | / |

**References**

1. Kekes, T., Kolliopoulos, G., Tzia, C., 2021.Hexavalent chromium adsorption onto crosslinked chitosan and chitosan/β-cyclodextrin beads: Novel materials for water decontamination.Journal of Environmental Chemical Engineering,9(4):100581.
2. Jia, D., Cai, H., Duan, Y., Xia, J., Guo, J., 2021.Efficient adsorption to hexavalent chromium by iron oxalate modified D301: Characterization, performance and mechanisms. Chinese Journal of Chemical Engineering,33:61-69.
3. Almeida, A., Nascimento, R., Amador, I., Santos, T., Martelli, M., Faria, L., Ribeiro, N., 2021. Chemically activated red mud: assessing structural modifications and optimizing adsorption properties for hexavalent chromium. Colloids and Surfaces A: Physicochemical and Engineering Aspects,628:127325
4. Aryee, A., Dovi, E., Li, Q., Han, R., Li, Z., Qu, L., 2022. Magnetic biocomposite based on peanut husk for adsorption of hexavalent chromium, Congo red and phosphate from solution: Characterization, kinetics, equilibrium, mechanism and antibacterial studies. Chemosphere,287(1):132030.
5. Liu, S., Gao, J., Zhang, L., Yang, Y., Liu, X., 2021. Diethylenetriaminepentaacetic acid–thiourea-modified magnetic chitosan for adsorption of hexavalent chromium from aqueous solutions. Carbohydrate Polymers,274:118555.
6. Luz-Asunción, M., Pérez-Ramírez, E., Martínez-Hernández, A., Castano, V., Sánchez-Mendieta, V., Velasco-Santos, C., 2019. Non-linear modeling of kinetic and equilibrium data for the adsorption of hexavalent chromium by carbon nanomaterials: Dimension and functionalization. Chinese Journal of Chemical Engineering,27(4):912-919.
7. Ajmani A, Shahnaz T, Subbiah S, Narayanasamy S (2019) Hexavalent chromium adsorption on virgin, biochar, and chemically modified carbons prepared from Phanera vahlii fruit biomass: equilibrium, kinetics, and thermodynamics approach. Environmental Science and Pollution Research 26:32137-32150
8. Niam AC, Fenelon E, Ningsih E, Mirzayanti YW, Kristanti E (2022) High-Efficiency Adsorption of Hexavalent Chromium from Aqueous Solution by Samanea saman Activated Carbon. Adsorption Science & Technology 2022:1-10
9. Yang J, Huang B, Lin M (2020) Adsorption of Hexavalent Chromium from Aqueous Solution by a Chitosan/Bentonite Composite: Isotherm, Kinetics, and Thermodynamics Studies. Journal of Chemical & Engineering Data 65:2751-2763
